# A comprehensive rabies vaccination program led by community pharmacists

**DOI:** 10.1177/17151635241286017

**Published:** 2024-10-28

**Authors:** Eddie T. Wong, Randy So, Fayaz Rajabali

**Affiliations:** Shoppers Drug Mart, Edmonton, AB; Shoppers Drug Mart, Edmonton, AB; Shoppers Drug Mart, Edmonton, AB

## Introduction

Community pharmacies receive weekly visits from 55% of Canadians (almost 10 times more frequently than their family physician), making pharmacies stand out as extremely accessible hubs for patients in need of health care services.^1,2^ Community pharmacists can offer clinical services to a wide range of patients due to their accessibility, particularly to those who are unable to see their family physician regularly. Furthermore, Alberta’s community pharmacists offer the most comprehensive range of services because their scope is the largest in the country, enabling them to better meet patients’ needs.^3,4^ Despite these advantages, it has been observed that not all community pharmacists are making the most of their abilities, which represents a possible lost opportunity given their demonstrated ability to improve patient outcomes.^5^

It is imperative for pharmacists to continually acquire new skills, enabling them to leverage their scope of practice for enhanced patient care. Pharmacists are in an advantageous position to relieve pressure on the health care system and serve as first-line health care providers, from initial assessment to comprehensive management and ongoing monitoring.

Our community pharmacy at Shoppers Drug Mart #317 has developed and implemented a rabies vaccination program that maximizes the use of our clinical services, enabling pharmacists to practise to their full scope. In collaboration with the Northern Alberta Institute of Technology (NAIT) and Alberta Health Services (AHS), we created a rabies vaccination program for students in both the Animal Health Technology (AHT) and Veterinary Medical Assistant (VMA) programs.

This program was needed as students studying in these fields required adequate protection against rabies due to their frequent exposure to animals. As our community pharmacy is located adjacent to NAIT, it was a perfect location for students to be able to access care conveniently during their studies. Through this program, we provided accessible care to students needing rabies immunization and followed through with them until they achieved adequate protection, which is normally defined as antibody titres of at least 0.5 IU/mL.^6^ However, NAIT requires students to achieve a value of at least 1.0 IU/mL to fulfill their immunization requirements, due to their continued potential exposure to this virus. We did not consult with NAIT regarding this requirement, as it is necessary to provide continuous protection for these students throughout their entire program, which typically lasts for 1 or 2 years. This heightened level of protection is reasonable to ensure their safety until they finish their program.

Rabies is a zoonic viral disease that works by infiltrating the host’s central nervous system. Rabies is typically spread by an infected animal’s saliva getting into wounds, generally through a bite.^6^ The fatality rate from rabies is around 100% if proper treatment is not received.^7^ Thus, it is crucial that people who come into contact with animals regularly, such as AHT or VMA students, be immunized to the rabies virus to be protected. The immunization protocol from Health Canada consists of either 3 intramuscular (IM) doses of 1.0 mL or 3 intradermal (ID) doses of 0.1 mL of rabies vaccine given on days 0, 7, and any time between days 21 and 28.^6^

We wanted to highlight how pharmacists can use their full scope of practice to enhance patient outcomes and reduce the burden on the health care system. As such, the objective of this study was to evaluate the impact of a community pharmacist–led rabies vaccination service in achieving adequate protection in AHT and VMA students.

## Methods

### Data extraction

We conducted a comprehensive retrospective chart review spanning the period from 2022 to 2023, focusing on AHT and VMA students who underwent rabies vaccination at Shoppers Drug Mart Pharmacy #317. We included all students in the AHT and VMA programs, which were identified by running a report through the Healthwatch Pharmacy Software for patients who were immunized with the rabies vaccine and then cross-referenced with previous appointment bookings to ensure we extracted data from the intended group. We excluded students who were immunocompromised, were taking immunosuppressive agents, had previous rabies vaccinations, or were currently pregnant.

### Rabies vaccine program recruitment

A registered nurse at NAIT facilitated the process for AHT and VMA students to receive their rabies vaccination at Shoppers Drug Mart Pharmacy #317. We organized 2 dedicated sets of days each year for students to schedule appointments for their vaccinations and informed them of the necessity to return for subsequent doses on day 7 and between days 21 and 28 following the initial appointment to complete the series.

At their appointment, the community pharmacist collected and assessed the student’s health information to ensure the rabies vaccine was indicated and safe for them. Both personal records and provincial electronic health records (Netcare) were used for assessment. The rabies vaccine used was RabAvert, an inactivated, purified chick embryo cell rabies vaccine, which is approved by Health Canada.^6^ The vaccines were provided from AHS, and there was no cost to these students. Students were only responsible for paying the injection administration fee.

### Rabies vaccination program

After the initial assessment of the students, the community pharmacist would counsel students on the vaccine route and schedule, and would then administer the rabies vaccine. We chose to administer a 0.1 mL ID dose using a 13 mm, 27G tuberculin syringe. This approach, administered at 0, 7, and 21 days, was chosen strategically to maximize the use of the vaccines provided by AHS, ensuring ample stock for all eligible students.

This is an approved vaccination route recommended by the World Health Organization as it uses less vaccine (1/10 of the IM dose) and provides similar protection.^7^ Vaccinations followed the Alberta College of Pharmacy guidelines to ensure proper and safe immunization procedures were followed.^8^

On the third vaccination, students were provided with a lab requisition form and instructed to visit the lab in 2 weeks so pharmacists could assess if an appropriate immune response was achieved. Lab results were obtained through the online provincial database (Netcare) or through mail. As noted above, a value of at least 0.5 IU/mL antibody titres is needed to protect against the rabies virus. However, NAIT requires students to achieve a value of at least 1.0 IU/mL to fulfill their immunization requirements. Students who did not achieve this value were offered a booster dose and provided with another lab requisition form to go for another blood draw 2 weeks after the booster dose.

This study was approved by the Health Research Ethics Board of Alberta—Community Health Committee. Waiver of consent was granted as no direct contact or interaction with patients occurred as a result of this study, and all identifiers were removed from the files prior to their review.

### Analysis

Data analysis was conducted by using descriptive statistics to analyze both demographic and clinical data. Statistical analysis was conducted using Microsoft Excel.

## Results

From the reports, *n* = 108 patient records satisfied both the inclusion and exclusion criteria. The mean age was 23 years (standard deviation 5.1; range, 17–47), with 94.4% (*n* = 102) of the sample population being female.

In terms of immune response, the mean titre levels observed were 5.74 IU/mL (standard deviation 1.75). The average time from the third vaccination to the blood draw, representing the waiting period, was 16.1 days (standard deviation 6.7).

According to the Canadian Immunization Guidelines (CIG), requiring at least 0.5 IU/mL antibody titres to be protected, 100% (*n* = 108) of the patients reached this level.^6^ In terms of the NAIT immunization requirements of antibody titres of at least 1.0 IU/mL, 99% (*n* = 107) of patients achieved this. This information is summarized in Table 1.

**Table 1:** Characteristics of AHT/VMA students participating in the rabies program

Characteristics	Rabies program patients (*n* = 108)
Male, *n* (%)	6 (5.6)
Female, *n* (%)	102 (94.4)
Age, mean (SD), y	23 (5.1)
Titres, mean (SD), IU/mL	5.74 (1.75)
Patients achieving titres of 0.5 IU/mL, *n* (%)	108 (100)
Patients achieving titres of 1.0 IU/mL, *n* (%)	107 (99.1)
Waiting period for blood draw, mean (SD), d	16.1 (6.7)
Patients with waiting period <14 days, *n* (%)	38 (35.2)

AHT, Animal Health Technology; IU, international units; SD, standard deviation; VMA, Veterinary Medical Assistant.

## Discussion

Our study demonstrates the success of a rabies vaccination program where community pharmacists play a pivotal role in both managing and providing care from start to finish. Community pharmacists used their full scope of practice to help patients (NAIT AHT/VMA students) reach their health goals.

One notable aspect of our program was the adoption of a cost-effective vaccine administration technique (ID), as we were able to cut costs for both patients and the health care system. Traditionally, the RabAvert vaccine is given as a 1.0 mL IM injection. However, by using a 0.1 mL ID injection, we were able to withdraw approximately 9 doses from the vial. A typical RabAvert vaccine would cost patients around $220 plus the pharmacist injection fee per dose (as this vaccine is not covered by the provincial government). However, by splitting the vaccine up, each dose now would cost around $25 plus the pharmacist injection fee, reducing the cost by nearly 90%. Considering the 3-dose schedule required for this vaccine, cost savings could equate to over $600 per patient. Indeed, this method of vaccination has been observed to have higher uptake, possibly due to reduced cost, while still providing adequate immune response.^9^ However, a drawback to this method is requiring multiple patients to be ready to receive the vaccine in a short period of time as the vaccine should be administered immediately after reconstitution.^10^

Serving as the primary health care provider for these patients, we demonstrated that community pharmacists can play a vital role in alleviating the pressing demands of the Canadian health care system. According to Statistics Canada, 4.7 million Canadians (14.4%) do not have a regular health care provider.^11^ For those that do have a health care provider, 41.7% of them would have to wait at least 3 days for an appointment.^11^ In light of these challenges, community pharmacists stand as accessible health care professionals capable of bridging the existing health care gap. Numerous studies have demonstrated significant improvements in health outcomes, reduction in costs to the health care system, and positive patient receptiveness when pharmacists practise to their full scope.^12-16^ By doing so, pharmacists can help address current health care disparities and create a more patient-centric approach to health care delivery.

To fulfill this crucial role effectively, pharmacists must leverage their full scope of practice. This ensures that they can provide comprehensive and uninterrupted care for patients, from the initial point of contact to the entirety of their health care journey. In the context of our rabies program, for instance, offering lab requisitions to patients for antibody titres becomes integral. If we did not provide lab requisitions to patients for antibody titres, patients would still need to seek out a general physician as part of their care, undermining the potential relief on the health care system that community pharmacists could otherwise provide. Therefore, community pharmacists must embrace their full scope of practice to ensure that care is provided uninterrupted.

Notably, 39 (35.1%) patients underwent lab testing earlier than the recommended 14 days according to the CIG.^6^ This recommendation is to ensure that the body has sufficient time to develop antibodies in response to the vaccine. Despite this, all patients in this subgroup achieved the target titres outlined in the CIG of >0.5 IU/mL and fulfilled their immunization requirement of titres >1.0 IU/mL set by NAIT.

For the lone patient who did not reach satisfactory titres following the vaccination program’s completion, an extra booster of the same vaccine was administered via intramuscular injection. Given that the vaccination program had ended (as most students receive their vaccination throughout September and October) and there was only 1 patient, the intramuscular route was preferred due to logistical constraints, as distributing multiple ID doses before expiration was impractical at that time. Following this additional dose, the patient attained titres of 3.64 IU/mL, fulfilling their NAIT immunization requirements successfully.

While acknowledging the limitation of a small sample size and the specific demographic characteristics of our study participants—mainly young and healthy individuals—we have observed a trend indicating that ID administration of the rabies vaccine elicits a rapid immune response. Indeed, these findings align with those from Kong et al.,^9^ who demonstrated a 99.9% seroconversion rate using the ID route, increased vaccination uptake, and reduced cost. Furthermore, when comparing seroconversion between the ID and IM route, a meta-analysis by Schnyder et al.^17^ found no differences between both groups and greater compliance in the ID group—possibly due to reduced costs.

Our preliminary findings suggest a promising aspect of the ID vaccination method in terms of its efficiency in triggering immune responses as well as cost-related savings attributed to the reduced vaccine volume required for each dose. Furthermore, our research indicates that pharmacists can implement this initiative and that their participation can improve the availability of this expensive vaccine.

## Conclusions

We have highlighted a program in which a pharmacist assumes the role of the primary health care provider, providing all-encompassing care from the very beginning. All patients acquired protection according to CIG guidelines, and 99% of patients fulfilled their school vaccination requirements. Beyond these successes, by reducing the need for appointments with physicians and maximizing vaccine usage, our program efficiently reduced time and expenses for patients as well as the health care system. This is only 1 example of how community pharmacists can help patients achieve their health goals and ultimately lessen the burden on the health care system by using their entire scope of practice. ■
